# Interplay of Oxidative Stress and Nitric Oxide Synthase Gene Expression on Cardiovascular Responses in Preeclampsia

**DOI:** 10.1055/s-0042-1742313

**Published:** 2022-01-31

**Authors:** Anita Herur, Manjunatha Aithala, Kusal K. Das, Ashalata Mallapur, Rajat Hegde, Suyamindra Kulkarni

**Affiliations:** 1 Department of Physiology, Shri B. M. Patil Medical College, Hospital and Research Centre, BLDE (Deemed to be University), Vijayapur-586103, Karnataka, India; 2 Department of Physiology and OBG, S. Nijalingappa Medical college, Bagalkot-587103, Karnataka, India; 3 Karnataka Institute for DNA Research (KIDNAR), Dharwad-580003, Karnataka, India

**Keywords:** molecular genetics, preeclampsia/eclampsia, vascular biology, genética molecular, pré-eclâmpsia/eclâmpsia, biologia vascular

## Abstract

**Objective** To assess the influence of oxidative stress on the gene expression of nitric oxide synthases (NOS 3 and NOS 2) and, hence, the cardiovascular responses in preeclampsia.

**Methods** This was a case control study in which patients with preeclampsia (PE group) and normal pregnancy controls (NP group) were included according to the guidelines of the American College of Obstetricians and Gynecologists (ACOG). The serum levels of malondialdehyde (MDA), total antioxidant capacity, and nitric oxide (NO) were estimated, and the heart rate and mean arterial pressure were recorded. The gene profiling of NOS3 and NOS2 was performed through real-time polymerase chain reaction (RT-PCR). The statistical analysis was performed using the Student *t* -test, and values of *p* < 0.05 were considered statistically significant.

**Results** The serum levels of malondialdehyde were increased ( *p* < 0.0001), and the total antioxidant capacity was reduced in the PE group ( *p* = 0.034), indicating oxidative stress. In the PE group, the mean arterial pressure was significantly higher ( *p* < 0.0001), but the serum levels of NO did not show a statistically significant reduction ( *p* = 0.20). The gene expression profiling of NOS3 and NOS2 revealed a down regulation in the PE group by 8.49 and 51.05 times respectively.

**Conclusion** Oxidative stress may lead to endothelial dysfunction, which could result in increased mean arterial pressure. Nitric oxide may play a role in this mechanism, but interactions with other vasoactive /biological substances cannot be overlooked, as the gene expression of NOS3 and NOS2 has been reduced.

## Resumo

**Objetivo** Avaliar a influência do estresse oxidativo na expressão genética das óxido nítrico sintases ( *nitric oxide synthases* , NOS, em inglês; NOS 3 e NOS 2) e, consequentemente, nas respostas cardiovasculares na pré-eclâmpsia.

**Métodos** Este foi um estudo caso-controle no qual pacientes com pré-eclâmpsia (grupo PE) e controles com gravidez normal (grupo GN) foram incluídos de acordo com as diretrizes do American College of Obstetricians and Gynecologists (ACOG). Foram estimados os níveis séricos de malondialdeído (MDA) da capacidade antioxidante total, e de óxido nítrico ( *nitric oxide* , NO, em inglês). A frequência cardíaca e a pressão arterial média foram registradas. O perfil genético da NOS3 e da NOS2 foi feito por reação em cadeia de polimerase em tempo real ( *real-time polymerase chain reaction* , RT-PCR, em inglês). A análise estatística foi feita utilizando-se o teste *t* de Student, e valores de *p* < 0,05 foram considerados estatisticamente significativos.

**Resultados** Os níveis séricos de malondialdeído sérico estavam aumentados ( *p* < 0,0001), e a capacidade antioxidante total, reduzida no grupo PE ( *p* = 0,034), o que indicava estresse oxidativo. No grupo PE, a pressão arterial média era significativamente maior ( *p* < 0,0001), mas os níveis séricos de NO não demostraram redução estatisticamente significativa ( *p* = 0,20). O perfil de expressão genética da NOS3 e da NOS2 revelou uma regulação negativa no grupo PE de 8,49 e 51,05 vezes, respectivamente.

**Conclusão** O estresse oxidativo pode levar à disfunção endotelial, o que pode resultar em aumento da pressão arterial média. O NO pode desempenhar um papel neste mecanismo, mas as interações com outras substâncias vasoativas/biológicas não podem ser negligenciadas, uma vez que a expressão genética da NOS3 e da NOS2 foi reduzida.

## Introduction

Normal pregnancy is marked by systemic inflammation, oxidative stress, and changes in angiogenic factors and vascular reactivity. This phenomenon is very much increased in pre-eclampsia (PE), with an impairment of compensatory mechanisms, eventually leading to vascular dysfunction. 1 The pathophysiology of PE is not completely understood. Nitric oxide (NO) may be responsible for gestational vasodilation due to its vasodilator action. 2 The production of NO from L-arginine is catalyzed by nitric oxide synthases (NOSs), which include neuronal NOS, endothelial NOS (eNOS/NOS3) and inducible NOS (iNOS/NOS2). 3

Reduced uterine perfusion pressure (RUPP) and placental ischemia may lead to endothelial and cardiovascular dysfunction through increased production of cytokines, which may trigger endothelial dysfunction by decreasing the bioavailability of NO and increasing that of reactive oxygen species (ROS). 4

The maternal vasculature is a major source of reactive oxygen and nitrogen species, which can interact to produce peroxynitrite, a powerful prooxidant that alters vascular function in PE. 1 Preeclampsia increases oxidative stress in the placental and maternal systemic circulations. 5

The expression of NOSs alters in PE; upregulation of eNOS expression has been demonstrated during normal pregnancy, 6 but the expressions of messenger ribonucleic acid (mRNA) and protein for eNOS are decreased in endothelial cells in cases of PE. 7

However, there is a dearth of literature highlighting the role of oxidative stress on NOS gene expressions and the impact of these on the systemic circulation. Hence, the present study aims to assess the influence of oxidative stress on NOS 3 and NOS 2 gene expressions and, hence, the cardiovascular responses in PE.

## Methods

This was a case-control study conducted in a tertiary care centre in North Karnataka, India. Clearance was obtained from the Ethics Committee at S. Nijalingappa Medical College, Bagalkot, Karnataka, India (SNMC/IECHSR/2017-18/A-48/1.1). Informed consent was obtained from all the participants. The size of the sample would have to be 23 participants in each group, which was calculated using the OpenEpi software, based on a study by Madazli et al., 2002 8 (the mean SOD between the groups was used), and 22 participants in each group, based on a study by Kashinakunti et al., 2010 9 (the mean MDA levels between the groups were used). Hence, the sample size was rounded off to 30 participants in each group.

Primigravidas aged between 18 and 35 years with singleton pregnancies and diagnosed with PE according to the guidelines of the American College of Obstetricians and Gynecologists (ACOG) 10 were included in the study as the cases (PE group). Pregnant women diagnosed with gestational hypertension or eclampsia according to the ACOG guidelines, and those with diabetes mellitus, history of systemic hypertension, and cardiovascular or renal diseases were excluded from the study.

Healthy primigravidas aged between 18 and 35 years with singleton pregnancies, matched for age and gestational week with the cases were included as controls (normal pregnancy [NP] group), and pregnant women with a history of diabetes mellitus, systemic hypertension, PE, and cardiovascular or renal diseases were excluded from the control group.

The required demographics were collected from the participants according to a predesigned proforma. They were clinically examined, and the findings were recorded. Gestational age was calculated from first trimester ultrasonography report and as per the ACOG guidelines. 11 Blood pressure was recorded using a mercury sphygmomanometer (Diamond, Maharashtra, India). The heart rate was caculated from lead II of the electrocardiogram (ECG), which was recorded using Powerlab (AD instruments, Sidney, Australia). The blood samples were drawn from the antecubital vein following aseptic precautions. Whole blood (2.5 mL) was transferred to PAXgene (QIAgen, Venlo, Netherlands) ribonucleic acid (RNA) tubes for NOS gene expression by real-time polymerase chain reaction (RT-PCR). Another 2.5 mL of blood was converted into serum for the estimation of the serum levels of NO (enzyme-linked immunosorbent assay [ELISA] kit method) and serum malondialdehyde (MDA, using the thiobarbituric acid reactive substance [TBARS] method), and the total antioxidant capacity (ELISA kit method).

### Gene Expression Profiling

Isolation of the total RNA from human whole blood was performed using the PAXGene RNA isolation kit and following the manufacturer instructions. The quality of the total RNA was checked by agarose gel (1.5%) electrophoresis, and the gel was observed with an ultraviolet (UV) transilluminator (Cleaver Scientific, Rugby, Warwickshire, United Kingdom). Complementary deoxyribonucleic acid (cDNA) was synthesised, and its quality was checked with agarose gel (1.5%) electrophoresis with a standardized protocol.

### Expression Profiling of NOS3

The gene expression profiling for NOS3 in samples from both the groups (NP and PE) was analysed using forward primer *5' CTGGCTTTCCCTTCCAGAT 3'* and reverse primer *5' CTTAATCTGGAAGGCCCCTC 3'* , along with the GAPDH gene as a positive control.

### Expression Profiling of NOS2

The gene expression profiling for NOS2 in samples from both the groups (NP and PE) was analysed using forward primer *5' GATATCCCCCAGCCCTCAAGT 3'* and reverse primer *5' GAGGCCCCAGTTTGAGAGAG 3'* , along with the Glyceraldehyde 3-phosphate dehydrogenase (GAPDH) gene as a positive control.

Each reaction was analysed in triplicate along with a negative control (without sample). Quantification cycles (Cq) values were recorded using the CFX Real-Time PCR (Bio-Rad, Hercules, CA, United States). The gene expressions of NOS3 and NOS2 were calculated using 2^ - ΔΔCq formula.

The serum levels of nitric oxide and total antioxidant capacity were estimated using ELISA kits as per the manufacturer's guidelines, and the serum levels of MDA were estimated by the TBARS method. 12

### Statistical Analysis

Data was analyzed with the Statistical Package for the Social Sciences (IBM SPSS Statistics for Windows, IBM Corp., Amonk, NY, United States) software, version 19.0, using the Student *t* –test, and values of *p* < 0.05 were considered statistically significant.

## Results

Table 1 depicts the mean age and the gestational age of the mothers in both groups, and the results showed that there was no significant difference between them.

**Table 1:** Maternal and gestational ages in normal pregnancy and preeclampsia

	Normal pregnancy (NP) group (n = 30)	Preeclampsia (PE) group (n = 30)	t-value	*p* -value
Maternal age (years)	22.97 ± 3.12	22.67 ± 3.83	0.333	0.215
Gestational age (weeks)	37.63 ± 3.16	36.10 ± 4.29	1.578	0.120

A significant oxidative stress was noted in the PE group by an increase in the serum levels of MDA and a decrease in total antioxidant capacity ( Table 2 ).

**Table 2:** Oxidative stress markers in normal pregnancy and preeclampsia

	Normal pregnancy (NP) group (n = 30)	Preeclampsia (PE) group (n = 30)	t-value	*p* -value
Serum malondialdehyde (nmol/mL)	2.50 ± 0.42	5.66 ± 1.02	12.682	0.0001
Serum total antioxidant capacity (ng/mL)	144.56 ± 65.24	112.37 ± 48.36	2.171	0.034

**Fig. 1.**
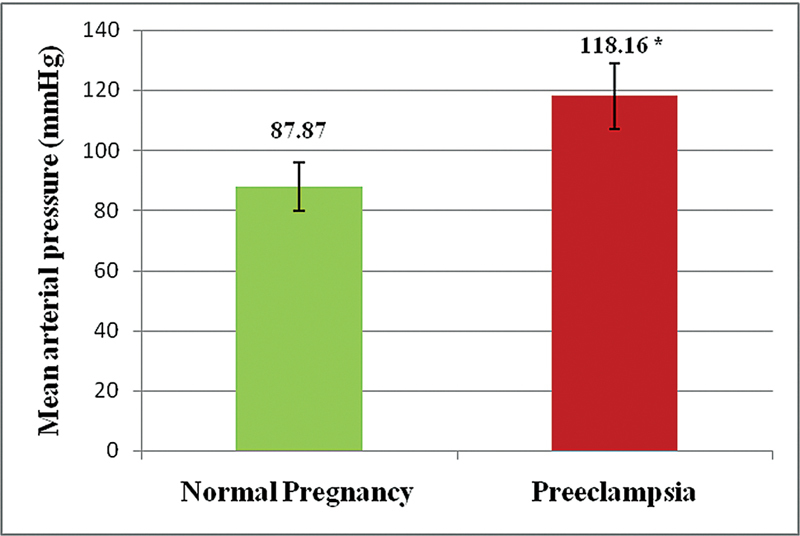
Mean arterial pressure of patients with preeclampsia.

The systolic, diastolic, and mean arterial pressures (in mmHg) were found to be significantly higher, by 37.26, 26.80, and 30.28 respectively in the PE group ( Table 3 ) ( Figure 1 ). The mean heart rate was slightly lower in the PE group as compared to the NP group, but not statistically significant.

**Table 3:** Cardiovascular responses in normal pregnancy and preeclampsia

	Normal pregnancy (NP) group (n = 30)	Preeclampsia (PE) group (n = 30)	t-value	*p* -value
Systolic blood pressure (mmHg)	116.80 ± 10.68	154.07 ± 17.10	10.122	0.0001
Diastolic blood pressure (mmHg)	73.40 ± 8.60	100.20 ± 8.64	12.044	0.0001
Mean arterial pressure (mmHg)	87.87 ± 8.19	118.16 ± 10.95	12.134	0.0001
Heart rate (beats/min)	93.10 ± 9.50	92.33 ± 12.04	0.274	0.785

There was no significant difference in the serum levels of NO in both the groups (NP group: 35.43 ± 20.37 μmol/L; PE group: 30.09 ± 10.08 μmol/L; t = 1.285; *p* = 0.204). The gene expression profiling was only performed in 25 suitable samples from each group. The mean difference in the Cq values of NOS3 in the RT-PCR, as against the positive control (GAPDH), was higher in the PE compared to the NP group. Consequently, when the gene expression of NOS3 was calculated, a down-regulation was observed in the PE group compared to the NP group by a difference of 8.49 times ( Table 4 ).

**Table 4:** Gene expression of endothelial nitric oxide synthase (NOS3) by real-time polymerase chain reaction

	Cq of the sample (NOS3) 1	Cq of the positive control (GAPDH) 2	ΔCq 1 - 2	ΔΔCq {Δcq of sample − average Δcq of control group}	2^ - ΔΔCq
Normal pregnancy group (n = 25)	23.14 ± 3.43	15.11 ± 1.82	7.96 ± 3.30	0.32 ± 3.63	14.63
Preeclampsia group (n = 25)	24.73 ± 2.56	15.63 ± 3.19	9.10 ± 2.74	1.14 ± 2.74	6.14

Abbreviations: Cq: Quantification cycles; GAPDH: Glyceradehyde 3-Phosphate dehydrogenase.

The mean difference in the Cq values of NOS2 in the RT-PCR as against the positive control (GAPDH) was higher in the PE group; consequently, when the gene expression of NOS2 was calculated, a down-regulation was oberved in the PE group compared to the NP group, by a difference of 51.05 times ( Table 5 ).

**Table 5:** Gene expression of inducible nitric oxide synthase (NOS2) by real-time polymerase chain reaction

	Cq of the sample (NOS2)	Cq of the positive control (GAPDH)	ΔCq	ΔΔCq {Δcq of sample − average Δcq of control group}	2^ - ΔΔCq
Normal pregnancy group (n = 25)	26.63 ± 1.79	16.47 ± 3.34	10.15 ± 3.49	0.004 ± 3.49	51.07
Preeclampsia group (n = 25)	27.40 ± 1.27	16.24 ± 2.59	11.16 ± 2.42	10.38 ± 3.19	0.02

Abbreviations: Cq: Quantification cycles; GAPDH: Glyceradehyde 3-Phosphate dehydrogenase.

## Discussion

Pregnancy is a state of oxidative stress due to increased metabolism in the mother and the metabolic activity of the placenta. The ischemic or poorly-perfused placenta resulting from inadequate trophoblast invasion exhibits increased oxidative stress in PE, evident from the increased formation of free radicals, lipid peroxides, and reduced antioxidant defenses. 1 13 The mean plasma levels of MDA were higher, and the levels of glutathione and superoxide dismutase were significantly lower in PE. 8 14 In the present study, we also observed an increase in the oxidative stress marker, serum MDA, which was accompanied by a decrease in the serum levels of total antioxidant capacity. In a study by Lee et al., 15 the neutrophils from women with PE produced significantly more ROS than those of the age-matched normotensive controls. Antioxidants protect against lipid peroxidation mediated by free radical. Normal pregnancy is associated with an increase in oxidative stress and lipid peroxidation, but antioxidant protection also increases. 16 In PE, there is still a further increase in lipid peroxides as well as an insufficient increase in antioxidants to combat the increase in oxidative stress and lipid peroxidation. 17 An increase in oxidative stress and a decrease in antioxidants may be responsible for the subsequent pathophysiology of PE. However, in one study, 18 supplementation with antioxidants like vitamins C and E did not show any difference in terms of reduction in the incidence of PE.

Reactive oxygen species seem to play an important role in the endothelial dysfunction associated with PE. Oxidative stress induces the adhesion of leukocytes and platelets to the endothelium as well as the release of cytokines and anti-angiogenic factors, which suggest an inflammatory state, as observed in PE.

Vascular endotheliosis, an increase in the inflammatory vasoconstrictors, and a decrease in the nitric oxide may have been responsible for an increase in the mean arterial pressure, as observed in the present study. A previous study 19 also revealed an increment of 13.3% in the mean arterial pressure in PE as against 5.2% in the normotensive group. But the decrease in serum NO was not statistically significant. Similarly, studies show an increase in NO production during pregnancy, and a decrease during PE, 3 or an increase in NO levels in PE, 20 or no change in NO levels. 21

Through the intrinsic synthesis of NO, eNOS is expressed constitutively in the vascular endothelium and maintains the vascular tone, hence inhibiting the adhesion of leukocytes and platelets to the endothelium and preventing the proinflammatory state. 22 Increased ROS production seems to suppress the expression and function of eNOS. 23 In the present study, a decrease in the expression of NOS3 was observed. Reduced expression of eNOS and oxidative stress could play a role in the pathology of PE, both in the placenta and in the maternal endothelium. 24 Increased ROS leads to lipid peroxidation, which increases the levels of MDA, as observed in the present study, and lipid peroxidation also decreases the calcium/adenosine triphosphatase (ATPase) activity, which in turn, may decrease the eNOS expression, as it is calcium dependent. 14

Oxidative stress leads to up-regulation of proinflammatory genes like iNOS, which is stimulated in a proinflammatory condition, such as NP, and produces a temporary excess of NO, maybe through activation of proinflammatory transcription factor nuclear factor kappa-light-chain-enhancer of activated B cells (NF-κB). 18 The literature also reports that the expression of iNOS is decreased, and that of eNOS is increased, as a result of the compensatory mechanism at a later stage in PE. 14 In the present study, the gene expressions of iNOS or NOS2, as well as of eNOS or NOS 3, were reduced. But our understanding is that a decrease in antioxidants as the cause has not been well supported, as supplementation of vitamins C and E failed to produce any positive response in PE. Hence, other mechanisms involved have to be contemplated. In PE, the inflammatory condition and excessive oxidative stress may stimulate the production of heat shock proteins (HSPs), which may suppress iNOS; and the role of VEGF, s-Flt or HIF-alpha should also be considered. The exact mechanisms underlying the pathophysiology of PE need to be researched in depth for the proper understanding, diagnosis, and therapeutic options. Although the gene expression was done in the present study, the quantification of the expression of proteins was not feasible with the available resources. A follow-up study on PE patients with serial NOS2 and NOS 3 gene expression is contemplated. The authors also intend to study the role of HSPs in PE.

## Conclusion

Oxidative stress may lead to endothelial dysfunction, which could result in increased mean arterial pressure, and NO may play a role in this mechanism, but interactions with other vasoactive /biological substances cannot be overlooked, as the gene expressions of NOS3 and NOS2 have been reduced.
